# Acceptance of mental illness and attitude towards pharmacotherapy among patients hospitalized in forensic psychiatry departments

**DOI:** 10.3389/fpsyt.2026.1800820

**Published:** 2026-04-30

**Authors:** Joanna Fojcik, Michał Górski, Daniel Szawarnoga, Anetta Lasek – Bal

**Affiliations:** 1 Department of Psychiatry, Department of Neurology, Faculty of Health Sciences in Katowice, Medical University of Silesia in Katowice, Katowice, Poland; 2 Department of Chronic Diseases and Civilization Threats, Department of Occupational Medicine and Hygiene, Faculty of Public Health in Bytom, Medical University of Silesia in Katowice, Katowice, Poland; 3 Department of Neurology, Faculty of Health Sciences in Katowice, Medical University of Silesia in Katowice, Katowice, Poland

**Keywords:** acceptance of illness, attitude, forensic psychiatry, isolation, pharmacotherapy

## Abstract

**Aim of the study:**

The aim of the study was to assess the level of acceptance of the disease and attitudes towards pharmacological treatment in patients hospitalized in forensic psychiatry departments and to analyze the relationship between these variables and the length of hospitalization.

**Materials and methods:**

The study included 121 patients hospitalized in forensic psychiatry wards. The Acceptance of Illness Scale (AIS) and the Drug Attitude Inventory (DAI) were used. Statistical analysis was performed using nonparametric tests, with a significance level of p < 0.05.

**Results:**

The mean AIS score was 28 points, indicating moderate to good disease acceptance. A positive attitude toward pharmacological treatment was demonstrated by 74% of respondents. There was no significant correlation between disease acceptance and attitudes toward treatment (p = 0.70), nor was there any effect of hospitalization length on attitudes toward pharmacotherapy (p = 0.317).

**Conclusions:**

Patients of forensic psychiatry wards demonstrate a medium or high level of acceptance of the disease and a mostly positive attitude towards pharmacotherapy; the lack of significant correlations between these variables and the independence from the length of hospitalization indicate the need for individualized therapy.

## 1 Entry

Patients hospitalized in forensic psychiatry units constitute a specific clinical population that coexists with chronic mental disorders, long-term hospitalization, and specific legal constraints related to the use of preventive measures. These factors can significantly influence the process of adaptation to the illness and attitudes toward pharmacological treatment, which remains the basis of treatment for psychotic disorders (1, 2).

Illness acceptance is one of the key indicators of psychological adaptation to a chronic condition. Higher levels of acceptance are associated with better emotional functioning, reduced negative reactions to the disease, and greater engagement in the therapeutic process (3). Attitudes toward pharmacological treatment, including beliefs about the efficacy and necessity of medication, play a significant role in shaping therapeutic cooperation and adherence (4, 5).

Previous research indicates that the relationship between illness acceptance and attitudes towards treatment is not clear-cut and may be modulated by clinical, social, and organizational factors within the healthcare system (6). In the population of forensic psychiatry patients, this issue remains relatively poorly understood.

The aim of this study was to quantitatively assess the level of acceptance of the disease and attitudes towards pharmacological treatment, as well as to analyze the relationship between these variables and the length of psychiatric hospitalization in patients of forensic psychiatry departments.

## 2 Materials and methods

### 2.1 Characteristics of the study group

The study involved patients hospitalized under the protective measures legally implemented in the Polish forensic psychiatry system. It was conducted in four forensic psychiatry centers. Recruitment period spanned from July to August 2025. A total of 150 patients meeting the eligibility criteria were identified, however, 29 patients refused to participate (19.3%), resulting in 121 participants (N = 121). Reasons for refusal were not analyzed. This decision stemmed from the need to maintain full voluntary participation without additional burden on patients with research procedures.

The average age of the respondents was 41 years (min. 19, max. 74). Men predominated in the group (79%). The most common marital status was single (64%), and the majority of respondents lived with their families (60%). The largest group consisted of people with secondary and vocational education, and the dominant form of professional activity was receiving a disability pension.

The largest group of patients in terms of length of stay in the ward were those hospitalized for less than a year (43% - 52). The next largest category were those who stayed in the ward for 1 to 3 years (30% - 36). Stays lasting 3 to 5 years accounted for 13% (16). 9% (11) of the study participants stayed in the ward for 5 to 9 years. The smallest group were patients hospitalized for over 9 years (5% - 6).

Among patients for whom information about the diagnosis was obtained (N = 116), paranoid schizophrenia predominated, diagnosed in 69% (80) of the study participants. Other diagnoses, including other psychotic disorders, were recorded in 31% (36) of the patients.

In the analyzed group of patients, various pharmacological treatment regimens were used, including both monotherapies and multi-component therapies.

Due to many small groups, the medication use was grouped into four groups: individuals taking antipsychotics (n = 85), individuals taking antipsychotics along with mood stabilizers (n = 31), individuals taking mood stabilizers alone (n = 2), and individuals not taking medication because they refused to take it (n = 3). Due to the extremely small numbers, the latter two groups were excluded from the comparisons.

sociodemographic characteristics of the study group are presented in **Table 1**.

**Table 1:** Sociodemographic characteristics of the study group (N = 121).

Specification		N	%	Comments
Age	19–29 years old	19	16%	average age: 41 years min: 19 years max: 74 years
	30–39 years old	44	36%	
	40–49 years old	35	29%	
	50–59 years old	13	11%	
	60–74 years old	10	8%	
Sex	woman	25	21%	
	man	96	79%	
Marital status	single	78	64%	
	in marriage	6	5%	
	after divorce	22	18%	
	lonely	15	12%	
Domicile	he lives alone	39	32%	
	lives with his family	72	60%	
	institutional care (ZOL, DPS)	10	8%	
Education	lack	2	2%	
	basic	15	12%	
	junior high school	24	20%	
	professional	31	26%	
	medium	38	31%	
	higher	11	9%	
Professional activity	works professionally	18	15%	
	unemployed	31	26%	
	pension	48	40%	
	work in sheltered conditions	2	2%	
	other	22	18%	

### 2.2 Study inclusion and exclusion criteria

The study inclusion criteria included the following factors:

- Diagnosis of a mental disorder
- Stable mental condition enabling participation in the study
- Status of a patient treated in judicial detention (preventive measure).
- Maintained ability to understand the content of the study and answer questions
- Expressing informed consent to participate in the study
- Typical drug therapy on the ward

The following factors were listed in the study exclusion criteria:

- Acute psychotic episode, manic episode or severe depression with psychotic symptoms.
- High intensity of negative symptoms preventing cooperation.
- Current suicidal thoughts or self-harming behavior requiring intervention.
- Cognitive deficits
- Inability to obtain informed consent.

Exclusion criteria were dictated by ethical considerations (the need to obtain informed consent), patient safety (current suicidal risk), and measurement reliability (the AIS and DAI require a preserved ability to reflect and understand the questions). Individuals excluded due to acute exacerbation of symptoms most often presented severe disorganized thinking and productive symptoms, significant severity of negative symptoms with withdrawal and limited communication, severe mood disorders with psychotic symptoms, significant cognitive deficits that hindered understanding of the research procedure, and current self-harming behavior or high suicidal risk.

Initial identification of potential participants was performed by the department’s medical staff (psychiatrist and nursing staff) based on inclusion and exclusion criteria, particularly current mental status and clinical stability. The role of the treatment team at this stage was limited solely to assessing medical eligibility.

Decision-making ability was assessed using a structured assessment based on the MacArthur tool Competence Assessment Tool (MacCAT -T/CR), encompassing understanding, appreciation, reasoning, and expressing choice. Capacity was assessed individually, not by diagnostic category, and no rigid cutoff point was used. In borderline cases, consent was deferred until clinical stabilization. This approach is consistent with recent systematic evidence demonstrating variability in decision-making capacity across schizophrenia spectrum and bipolar disorder across domains and stages, emphasizing that diagnosis alone does not determine capacity (7).

The actual process of informing about the study and obtaining informed consent was conducted by a member of the research team not involved in the patient’s current treatment or in preparing forensic psychiatric opinions.

The ability to provide informed consent was assessed individually by a psychiatrist before the offer of participation in the study. Specifically, the ability to understand information about the study, the ability to logically consider alternatives, and the ability to express a stable and voluntary decision were assessed.

If there were any doubts about the patient’s current decision-making capacity, the patient was not eligible to participate in the study.

To reduce the risk of perceived coercion, those responsible for treatment and research consent held separate roles. Furthermore, patients received clear information about the voluntary nature, purpose, and nature of the study. Patients also had the opportunity to ask questions in a confidential setting and to withdraw consent at any time—without having to provide a reason and without any consequences.

### 2.3 Research tools

The study used:

3.1 Clinical and Demographic Data Questionnaire - The analysis of clinical data included two variables collected in a complete manner: the length of the current psychiatric hospitalization and the pharmacotherapy used, in particular the scope and type of neuroleptic therapy. One of the clinical variables that was incomplete was the psychiatric diagnosis. Due to the significant role of the diagnosis in the context of the study, the data were subjected to multiple imputation to complete the data.

3.2 The Acceptance of Illness Scale (AIS) – a tool for assessing the degree of adaptation to chronic illness, consisting of eight items rated on a five-point scale, with a total score ranging from 8 to 40 points (3). Higher scores indicate better adaptation to the illness, a reduced sense of discomfort, and a higher level of acceptance of one’s own limitations. Scores ranging from 8 to 18 indicate a low level of acceptance, characteristic of significant adaptation difficulties and a strong sense of burden of the illness. The range of 19 to 29 points corresponds to a medium level, reflecting moderate psychological adaptation. Values ranging from 30 to 40 indicate a high level of acceptance, associated with good adaptation to the illness and a relatively small impact of health limitations on emotional functioning. This approach allowed not only for the assessment of the overall level of illness acceptance in the study group of patients but also for the analysis of the variation in the intensity of this process, which forms the basis for further quantitative studies. No missing data were recorded. Cronbach’s alpha calculations for the AIS tool (α = 0.85) indicate a satisfactory level of reliability.

3.3 Drug Attitude Inventory (DAI) – assessed using a tool designed to measure individual beliefs, experiences, and patient attitudes toward pharmacotherapy. The questionnaire consists of thirty statements scored dichotomously (YES/NO), with each item assigned a response key specifying whether it indicates a pro-drug (P) or anti-drug (F) attitude. According to the scoring system, responses consistent with a positive attitude toward treatment receive +1 point, while responses inconsistent with it receive -1 point. The overall score ranges from -30 to +30, with positive values indicating a positive attitude toward pharmacotherapy, 0 indicating a neutral attitude, and negative values indicating a negative attitude (4). A complete data set was collected, with no missing values. The Kuder-Richardson 20 index demonstrated high reliability (KR-20 = 0.83).

### 2.4 Statistical analysis

The collected data were initially verified for completeness and logical accuracy of the responses. The analysis was quantitative in nature.

Normality of distribution was assessed for all numerical variables using the Shapiro -Wilk and Lilliefors tests. For variables that did not meet the assumptions

of normality, appropriate nonparametric tests were applied.

The research hypotheses were verified using the following procedures:

− Spearman’s rank correlation test with 95% confidence interval determined using the BCa method – to assess the relationships between variables for which a normal distribution was not found,
− chi-square test of independence and a variant of exact tests: for 2x2 tables, Fisher’s exact test, for tables larger than 2x2, the Fisher-Freeman-Halton exact test – to examine the dependencies between nominal and ordinal variables,
− F test) – in the case of comparisons between groups when the assumptions regarding the distribution are met,
− Kruskal – Wallis test – as an alternative to the analysis of variance in the absence of a normal distribution,
− Mann Whitney *U* test – for comparisons of two groups in terms of variables for which there was no normal distribution and numerical disproportion between groups,
− linear regression analysis to test the association while controlling for covariates.

The level of statistical significance was set at α = 0.05. Both quantitative and qualitative variables were presented in tabular and graphical forms (tables and graphs).

For quantitative variables, arithmetic means (M), standard deviations (SD), and medians (Me) were additionally calculated. Frequencies and percentages were presented for all analyzed variables, with percentages rounded to integers for clarity.

The analysis also included cases with incomplete clinical data regarding psychiatric diagnosis and year of first schizophrenia diagnosis. Due to significant missing data (<60%), analysis of year of diagnosis was abandoned, but analysis of missing data for diagnosis was undertaken.

Statistical analysis was performed using Microsoft Excel and IBM SPSS Statistics (version 29.0, IBM Corp., Armonk, NY, USA). The study received a positive opinion from the Bioethics Committee of the Medical University of Silesia in Katowice, BNW/NWN/0052?KB/94/25. The study was conducted in accordance with the principles of the Declaration of Helsinki and applicable legal regulations regarding research involving hospitalized individuals.

## 3 Results

### 3.1 Acceptance of the disease

The mean score on the Illness Acceptance Scale was 28 points, with a range of 8 to 40. Low levels of illness acceptance were found in 11% of the study participants, medium levels in 47%, and high levels in 42% of patients. These results indicate significant variation in the degree of adaptation to illness within the study population.

The Shapiro -Wilk test result was statistically significant: *W* = 0.96; *p* = 0.003, which indicates a violation of the normality assumption. However, the results were insignificant when divided by the duration of hospitalization (group < 1 year: *W* = 0.96; *p* = 0.053; 1–3 years: *W* = 0.95; *p* = 0.127; > 3 years: *W* = 0.95; *p* = 0.119). A distribution similar to normal was observed among patients treated with antipsychotics and stabilizers (*W* = 0.94; *p* = 0.072), but skewed among those treated only with antipsychotics (*W* = 0.95; *p* = 0.003).

### 3.2Attitudes towards pharmacological treatment

Scores in the study group ranged from -18 to 28 points. The mean score was

M = 9.59 (SD = 11.43), while the median Me = 12, indicating that the majority of patients had positive scores, characteristic of a positive attitude toward treatment. The histogram of the distribution shows a predominance of positive values with a relatively small number of strongly negative scores, confirming the prevalence of a positive attitude toward pharmacotherapy in the forensic psychiatry ward population.

After classifying the results into three categories, it was found that the vast majority (74% - 90) of patients had a positive attitude toward pharmacotherapy. A neutral attitude was noted in 6% (7) of respondents, while 20% (24) of study participants had a negative attitude.

The Shapiro -Wilk test result for attitude was also statistically significant: *W* = 0.96; *p* = 0.002. When divided by duration of hospitalization, a significant result was noted in the 1–3 years group (*W* = 0.92; *p* = 0.015), the remaining results were insignificant (group < 1 year: *W* = 0.96; *p* = 0.113; group > 3 years: *W* = 0.96; *p* = 0.295). Significant test results were reported depending on the treatment regimen (antipsychotics: W *=* 0.96; *p* = 0.019; antipsychotics with stabilizers: *W* = 0.86; *p* < 0.001).

### 3.3 Diagnosis of missing data

Analysis of missing data revealed their presence only for the variable “diagnosis” (n = 5; 4.13%). Complete data were available for 116 individuals (95.87%), of whom 80 (66.12%) were diagnosed with paranoid schizophrenia, and 36 (29.75%) had another diagnosis. It was decided to supplement the missing data due to the potential significance of this variable as a predictor in the analyses.

In order to assess the mechanism of missing data, comparative analyses were performed between individuals with missing data and individuals with complete data for all variables included in the study. The Mann- Whitney U test was used for quantitative variables, and the chi-square test or its exact equivalents for nominal and ordinal variables. In most cases, no statistically significant differences were found (p > 0.05). The only significant relationship was noted for the treatment regimen in the Fisher-Freeman-Halton exact test: χ²(2) = 12.32; p = 0.007; Vc = 0.51. Analysis of column proportions showed no significant differences for the use of antipsychotics, antipsychotics with mood stabilizers, and mood stabilizers. However, a significant difference was found in the category of missing medication (group with missing data: n = 2; 40%; group with complete data: n = 1; 0.9%). This may indicate the potential occurrence of the MAR (Missing at Random), i.e. the dependence of missing data on the treatment regimen.

Therefore, missing data were addressed using multiple imputation using fully conditional specification (FCS), generating five imputed datasets. The imputation model included all variables included in the analyses, and a logistic regression model was used for imputation. Final estimates were combined according to Rubin’s rules.

The distribution of diagnoses in subsequent imputed data sets was similar and was as follows (paranoid schizophrenia vs. other diagnosis):

1. 84 (69.4%) vs 37 (30.6%),
2. 83 (68.6%) vs 38 (31.4%),
3. 81 (66.9%) vs 40 (33.1%),
4. 80 (66.1%) vs 41 (33.9%),
5. 83 (68.6%) vs 38 (31.4%).

For the combined estimate, the mean sample size was 82.2 vs. 38.8, respectively. To assess the stability of the results, a sensitivity analysis was also performed for linear regression models. The analyses also accounted for missing data regarding treatment regimen, which did not overlap with missing data in the diagnosis variable.

### 3.4 Differences in disease acceptance and attitude towards treatment depending on the treatment regimen

We decided to examine whether the treatment regimen differentiated the level of illness acceptance and attitude toward pharmacological treatment. Due to many small groups, we decided to combine the medication intake into four groups: individuals taking antipsychotics (n *=* 85), individuals taking antipsychotics along with mood stabilizers (*n* = 31), individuals taking mood stabilizers alone (*n* = 2), and individuals not taking medication (*n* = 3). Due to the extremely low numbers, the latter two groups were excluded from comparisons. Analysis using the Mann -Whitney *U test* revealed a statistically insignificant result for illness acceptance: *Z* = -0.87; *p* = 0.377; *r*_rb_ = -0.11 [-0.33; 0.13], but a significant result for attitude toward pharmacological treatment: *Z* = -2.00; *p* = 0.046; *r*_rb_ = 0.24 [0.01; 0.45]. The observed effect was weak, however, the analysis of mean ranks and medians shows that the respondents taking antipsychotics together with mood stabilizers had a more positive attitude towards pharmacological treatment (*Me* = 16.00) than the respondents taking antipsychotics alone (*Me* = 8.00).

**Table 2** presents the detailed results of the analysis.

**Table 2:** Results of the Mann -Whitney U test for AIS and DAI depending on the treatment regimen (N = 116).

	Antipsychotic drugs (*n* = 85)			Antipsychotic drugs and mood stabilizers (*n* = 31)					
Variables	Average rank	*Me*	*IQR*	Average rank	*Me*	*IQR*	*WITH*	*p*	*r*_rb_ [95% *CI]*
AIS	60.13	28.00	9.00	54.03	29.00	14.00	-0.87	0.387	-0.11 [-0.33; 0.13]
DAI	54.74	8.00	18.00	68.82	16.00	14.00	-2.00	0.046	0.24 [0.01; 0.45]

### 3.5 The relationship between acceptance of the disease and attitudes towards treatment

Spearman’s rank correlation coefficient was used to analyze the relationship between the Acceptance of Illness Scale (AIS) score and attitude toward pharmacological treatment (DAI) in the study group of patients. Due to potential outliers and skewed distributions, the analysis was based on the bootstrapping method _with_ a sample size of 5000 using the BCa method. The analysis results did not indicate a statistically significant correlation between the variables: *rs* = -0, 04 [-0.21; 0.16]; *p* = 0.700. Based on the obtained results, there is no basis to reject the null hypothesis that there is no relationship between the AIS score and attitude towards pharmacological treatment. The alternative hypothesis postulating the existence of such a relationship was not confirmed.

### 3.6 Differences in disease acceptance and attitude towards treatment depending on the length of hospitalization

To assess whether attitudes toward pharmacotherapy and illness acceptance differed depending on the duration of hospitalization, DAI and AIS scores were compared across three hospitalization length ranges: up to 1 year, 1 to 3 years, and over 3 years. Due to the non-normal distribution of the DAI variable, the Kruskal - Wallis test was used, but a one-way ANOVA was performed for the AIS. The analysis revealed no statistically significant differences between the study groups for DAI: *H* (2) = 2.30; *p* = 0.317; η ^2^ < 0.01 [0.00; 0.12]. The highest median was obtained among patients hospitalized for 1 to 3 years (Me = 14), while the lowest was in the group staying in the ward for over 3 years (Me = 8). However, these differences were not statistically significant.

Additionally, differences in AIS were checked, but they were also statistically insignificant: *F* (2, 118) = 0.59; *p* = 0.559; η ^2^ = 0.01 [0.00; 0.06].

The results suggest that the length of psychiatric hospitalization did not significantly differentiate DAI and AIS scores. Patients’ attitudes toward pharmacotherapy and illness acceptance were not related to the length of stay in the hospital, and any differences may be due to other clinical factors or patients’ personal experiences.

The data are presented in **Table 3**.

**Table 3:** Results of the Kruskal- Wallis test with one-way ANOVA for DAI and AIS depending on the duration of hospitalization (*N* = 121).

Variable	Up to 1 year (*n* = 52)			From 1 to 3 years (*n* = 36)			Over 3 years (*n* = 33)			*H* (2)/ *F* (2, 118)	*p*	η ^2^ [95% *CI]*
	*M*	*SD*	*Me* (*min* - *max)*	*M*	*SD*	*Me* (*min* - *max)*	*M*	*SD*	*Me* (*min* - *max)*			
DAI	9.04	11.49	10 (-14 - 28)	11.61	12.17	14 (-16 - 28)	8.24	10.53	8 (-18 - 24)	2.30	0.317	<0.01 [0.00; 0.12]
AIS	27.33	7.78	27 (8-40)	29.14	7.24	29.50 (8-40)	28.03	8.16	28 (11-40)	0.69	0.559	0.01 [0.00; 0.06]

### 3.7 Analysis of AIS and DAI predictors

In order to check the differences in the AIS and DAI, calculations were performed based on a multivariate model: linear regression analysis. First, analyses were performed for the AIS. The model included age, gender (coding: 1 – female, 2 – male), treatment regimen (coding: 1 – antipsychotic drugs, 2 – antipsychotic drugs and stabilizers), length of hospitalization (dummy included). coding, with hospitalization time below one year as the reference level), diagnosis (coding: 1 – paranoid schizophrenia, 2 – other). The analysis took into account missing data (*n* = 5) resulting from too small treatment regimen groups. The source data were taken into account, as well as data after imputation within the diagnosis (**Table 4**).

**Table 4:** Results of linear regression analysis for AIS based on sociodemographic and treatment-related variables .

Variables	Sample *N* = 113				Sample *N* = 116		
	*B*	95% *CI*	β	t	*B*	95% *CI*	*t*
Constant	25.43	15.63-35.22		5.15***	26.24	16.44-36.04	5.25***
Age	-0.03	-0.15-0.09	-0.05	-0.52	-0.05	-0.17-0.07	-0.87
Sex	3.56	0.12-6.99	0.20	2.05*	3.65	0.22-7.08	2.08*
Treatment regimen	-2.91	-6.19-0.36	-0.18	-1.76	-2.82	-6.09-0.44	-1.70
LOS (ref. < 1 year)							
1–3 years	2.70	-0.71-6.12	0.17	1.57	2.36	-0.98-5.71	1.38
> 3 years	1.59	-1.90-5.07	0.10	0.90	1.37	-2.12-4.86	0.77
Diagnosis	-0.14	-3.25-2.98	-0.01	-0.09	-0.11	-3.23-3.02	-0.07
Model	*F* (6, 106) = 1.52; *p* = 0.180; *R* ^2^ = 0.079				*F* (6, 109) = 1.57; *p* = 0.162; *R* ^2^ = 0.080		

*B*, unstandardized regression coefficient; *CI*, confidence interval; β, standardized regression coefficient; *t, Student’s t-* test value; *F*, ANOVA test result; *R* ^2^, coefficient of determination; Hospitalization time was coded using the dummy method coding, and hospitalization duration up to one year was set as the reference level. Gender coding: 1, female, 2, male. Treatment regimen coding: 1, antipsychotics, 2, antipsychotics and stabilizers; those treated only with stabilizers (*n* = 2) or without treatment (*n = 3)* were excluded. Diagnosis coding: 1, paranoid schizophrenia, 2, other. The beta coefficient was not generated for variables after imputation.

*p < 0.05; **p < 0.01; ***p < 0.001.

The model for the source data turned out to be an inadequate fit: *F* (6, 106) = 1.52; *p* = 0.180 and explained 8% of the variability in AIS. It is worth noting that only gender was a significant predictor, and the value of the coefficient would indicate better acceptance of the illness in men than in women. Similarly, a non-significant ANOVA result was noted for the model after data imputation: *F* (6, 109) = 1.57; *p* = 0.162. The percentage of explained variance did not change (8%), nor did the values of the predictors.

Next, the model for DAI was tested. In this case, the model was built hierarchically, initially entering control variables along with hospitalization time and diagnosis, followed by AIS (**Table 5**).

**Table 5:** Results of linear regression analysis for DAI based on sociodemographic, treatment-related and disease acceptance-related variables (N = 113/116).

Variables	Sample *N* = 113								Sample *N* = 116					
	*B*	95% *CI*	β	*t*	*B*	95% *CI*	β	*t*	*B*	95% *CI*	*t*	*B*	95% *CI*	*t*
Constant	16.53	1.48-31.59		2.18*	14.93	-1.97-31.83		1.75	16.31	1.37-31.26	2.14*	15.54	-1.24-32.32	1.82
Age	-0.11	-0.29-0.08	-0.11	-1.11	-0.10	-0.29-0.09	-0.10	-1.08	-0.09	-0.27-0.09	-0.94	-0.09	-0.27-0.10	-0.92
Sex	-4.19	-9.47-1.10	-0.15	-1.57	-4.41	-9.82-1.00	-0.16	-1.62	-4.71	-9.91-0.49	-1.78	-4.82	-10.15-0.51	-1.77
Diagram treatment	4.93	-0.10-9.96	0.19	1.94	5.11	-0.01-10.24	0.20	1.98	5.34	0.40-10.29	2.12*	5.42	0.39-10.46	2.11*
LOS (ref. < 1 year)														
1–3 years	1.72	-3.53-6.97	0.07	0.65	1.55	-3.78-6.88	0.06	0.58	1.20	-3.86-6.27	0.47	1.13	-4.00-6.26	0.43
> 3 years	-2.25	-7.61-3.11	-0.09	-0.83	-2.35	-7.75-3.05	-0.09	-0.86	-2.14	-7.42-3.15	-0.79	-2.18	-7.50-3.15	-0.80
Diagnosis	-0.63	-5.42-4.15	-0.03	-0.26	-0.63	-5.43-4.18	-0.03	-0.26	-0.82	-5.66-4.03	-0.33	-0.81	-5.68-4.06	-0.33
AIS	–	–	–	–	0.06	-0.23-0.36	0.04	0.42	–	–	–	0.03	-0.26-0.32	0.20
Model	*F* (7, 105) = 1.31; *p* = 0.253; *R* ^2^ = 0.080								*F* (7, 108) = 1.30; *p* = 0.257; *R* ^2^ = 0.078					
Change	ΔF (*1*, 105) = 0.18; *p* = 0.675								ΔF (*1*, 108) = 0.04; *p* = 0.840					

*B*, unstandardized regression coefficient; *CI*, confidence interval; β, standardized regression coefficient; *t*, Student’s *t- test value*; *F*, ANOVA test result; *R* ^2^, coefficient of determination; Δ *F*, change in *F*; Δ *R* ^2^, change *in R* ^2^. Hospitalization time was coded using the dummy method coding, and hospitalization duration up to one year was set as the reference level. Gender coding: 1, female, 2, male. Treatment regimen coding: 1, antipsychotics, 2, antipsychotics and stabilizers; those treated only with stabilizers (*n* = 2) or without treatment (*n = 3)* were excluded. Diagnosis coding: 1, paranoid schizophrenia; 2, other. The beta coefficient was not generated for variables after imputation.

Again, the model on the original data showed no fit: *F* (7, 105) = 1.31, *p* = 0.253. The metric, treatment, and AIS variables together explained 8% of the variance in DAI, of which the addition of AIS to the model did not improve it. None of the predictors was statistically significant.

The model on imputed data also did not fit well: *F* (7, 108) = 1.30; *p* = 0.257. The model also explained 8% of the variance in DAI. In this case, the treatment regimen was a significant predictor, and individuals treated with antipsychotics combined with mood stabilizers had a more positive attitude toward pharmacological treatment than those treated with antipsychotics alone. However, it is important to note the poor fit of the models for both the AIS and DAI, which weakens the interpretative power of the significant predictors. However, the models indicate that sociodemographic and treatment-related factors were not a good choice for explaining the variance in these variables. There was also no change in the relationship between illness acceptance and attitude toward treatment when controlling for controlled variables.

## 4 Discussion

The aim of this study was to assess the level of illness acceptance and attitudes toward pharmacological treatment in patients hospitalized in forensic psychiatry units, as well as to analyze the relationship between these variables and the length of psychiatric hospitalization. The obtained results indicate significant variation in the psychological functioning of the study group and a lack of simple, unambiguous relationships between the analyzed constructs.

In this study, most patients had AIS scores in the moderate to high range, suggesting moderate to good adjustment to mental illness. This finding is partially consistent with the observations of other authors who indicate that in patients with chronic mental disorders, illness acceptance may increase with duration of treatment, stabilization of symptoms, and adaptation to institutional settings (1, 2).

At the same time, it is important to emphasize that the study sample also included a group of patients (11%) with low levels of illness acceptance, suggesting persistent adaptation difficulties despite prolonged hospitalization. These individuals may experience increased frustration, illness denial, reduced motivation for treatment, and an increased risk of interpersonal conflict. In clinical practice, this group should be identified as requiring more intensive psychological interventions aimed at supporting the adaptation process and reducing the subjective burden of illness.

The literature emphasizes that illness acceptance in psychotic disorders is not a simple function of the duration of the illness, but depends on factors such as the level of insight, experience of stigmatization, interpersonal relationships, and subjective sense of agency (3–5). The specific situation of forensic psychiatric patients – related to limited autonomy and the compulsory nature of treatment – may additionally modulate this process.

Analyzing the study data, we conclude that most patients achieve scores within the average or high range, which may indicate relatively stable mental functioning in the context of a chronic illness. However, it should be emphasized that the study included only patients in a state of relative mental stability. Therefore, the obtained results pertain primarily to this group of patients and cannot be directly generalized to patients admitted with an acute mental state or during a phase of symptom exacerbation. In the case of individuals experiencing an acute episode of illness, the level of psychological adaptation, insight into the illness, and attitudes toward treatment may vary. At the same time, the presence of a group of individuals with low levels of illness acceptance emphasizes the need for an individualized therapeutic approach focused on adaptive support and reducing mental burden.

The vast majority of respondents (74%) presented a positive attitude towards pharmacological treatment, which is confirmed by the results of previous studies conducted in populations of patients diagnosed with schizophrenia and other psychotic disorders treated in inpatient settings (6, 8). A positive attitude towards pharmacotherapy is sometimes interpreted as a result of long-term clinical stabilization, experiencing symptom reduction, and adaptation to the treatment routine (9).

At the same time, it is clinically significant that one in five patients presented a negative attitude toward pharmacotherapy. According to current reports, negative attitudes toward medications may be related to experiencing adverse effects, a lack of control over therapeutic decisions, and reduced insight into the illness (10, 11). In the forensic psychiatric population, these factors may be further exacerbated by coercive treatment and limited ability to negotiate the pharmacotherapy regimen. This finding indicates that although most patients accept pharmacotherapy and perceive its benefits, a significant minority demonstrate difficulty accepting treatment, which may clinically be associated with the risk of irregular medication administration or reduced therapeutic compliance.

Recent research on the therapeutic relationship and communication in medicine shows that, under legal and systemic pressure, physicians are increasingly adopting so-called “defensive psychiatry.” This means taking action primarily to mitigate legal risk, rather than always with the patient’s best interests in mind. In practice, this can weaken the doctor-patient relationship and limit the patient’s participation in treatment decisions. Such systemic conditions impact clinical communication: physicians may avoid openly discussing patient concerns about pharmacotherapy, which patients perceive as a lack of alliance and autonomy (12).

Analysis of the DAI results indicates that patients in the forensic psychiatry ward overwhelmingly hold a positive attitude toward pharmacotherapy, as confirmed by both the mean values and the distribution of the three attitude categories. At the same time, the presence of a group of individuals with a negative attitude emphasizes the need for individual work on motivation, psychoeducation, and monitoring of therapeutic adherence.

One of the key findings of this study is the lack of a statistically significant relationship between the level of illness acceptance (AIS) and attitudes toward pharmacological treatment (DAI). This result can be interpreted as consistent with the view, increasingly expressed in the literature, that illness acceptance and attitudes toward treatment may be, to some extent, independent constructs (10, 13).

Research indicates that patients may accept the fact of their illness as a part of their lives, yet simultaneously hold ambivalent or negative beliefs about pharmacotherapy, particularly in the context of concerns about the long-term consequences of treatment (14). On the other hand, a positive attitude toward medications may result from a pragmatic approach to treatment, even with a low level of acceptance of the illness itself. The results of this study support the thesis that adaptation processes and attitudes toward pharmacotherapy have distinct psychological and clinical determinants.

The results indicate that in the study sample, the AIS score is not associated

with beliefs about pharmacotherapy. Attitudes toward medication use may be shaped by other factors, such as previous therapeutic experiences, the quality of relationships with medical staff, or individual levels of insight into the illness, rather than by the Illness Acceptance Scale score alone. It is worth emphasizing that insight into the illness is a multidimensional construct. It encompasses not only understanding and acceptance of the diagnosis but also awareness of the need for treatment and willingness to cooperate with therapy, both psychologically and pharmacologically. Therefore, the level of insight may be a significant intermediary between the severity of psychopathological symptoms and attitudes toward treatment.

The literature also reports studies indicating a correlation between the level of illness acceptance and attitudes toward pharmacological treatment. These relationships are explained, among other things, by the influence of common factors, such as the level of insight into the illness, the experience of symptoms, and the quality of the therapeutic relationship (15). At the same time, it is emphasized that these constructs are not identical – illness acceptance refers primarily to psychological adaptation to a chronic condition, while attitudes toward treatment concern beliefs and experiences directly related to pharmacotherapy. As a result, both areas may demonstrate partial interdependence, but at the same time remain relatively independent, and in certain clinical conditions – including the forensic psychiatric population – their mutual relationship may weaken. Similar conclusions are presented by the authors of studies on therapeutic adherence, indicating that attitudes toward pharmacotherapy are shaped not only by illness acceptance but also by treatment-related experiences, perceived side effects, and a sense of influence on therapeutic decisions (16).

In the full dataset, significant variation was observed in the duration of patients’ stays in the forensic psychiatry ward. The wide variation in hospitalization duration reflects the specific nature of forensic psychiatry wards, where the length of treatment depends both on the dynamics of mental illness symptoms and on legal conditions, including court decisions regarding the use of preventive measures.

The lack of a significant relationship between length of hospitalization and attitudes toward pharmacological treatment is consistent with research indicating that length of stay alone is not a sufficient predictor of therapeutic attitudes (17, 18). The literature increasingly emphasizes the quality of the therapeutic relationship, the availability of psychoeducation, and the individualization of treatment, rather than the length of hospitalization itself (19, 20). From a clinical perspective, this emphasizes the importance of the quality of therapeutic interactions, not their duration. It should also be noted that attitudes toward pharmacotherapy may change depending on the stage of treatment and the patient’s degree of adaptation to the therapeutic process. In the initial phases of hospitalization, especially when treatment is compulsory, patients may demonstrate greater ambivalence or resistance to pharmacotherapy, which may change over time as their mental state improves and treatment effectiveness is experienced. The consistent availability of psychoeducation, the cohesion of the therapeutic team, and the patient-staff partnership may have a greater impact on patient attitudes than the number of years spent in hospitalization.

In forensic psychiatry, the length of stay is often determined by legal factors, not solely clinical ones, which may further weaken its relationship with the psychological aspects of adaptation to illness and treatment. Pharmacotherapy is sometimes conducted under conditions of limited patient autonomy, and the duration of hospitalization also depends on the court’s decision. These factors may influence attitudes toward treatment, the sense of agency, and the therapeutic relationship. This approach is consistent with the literature indicating that in forensic psychiatry, the recovery process and therapeutic cooperation are largely shaped by institutional and legal conditions (19, 21, 22).

It should be noted that legal arrangements regulating the functioning of forensic psychiatry vary across jurisdictions, which may modify the scope and manner of these factors’ impact. Furthermore, in this study, legal and institutional variables were not directly operationalized or empirically analyzed; therefore, they are treated as an interpretive context for the obtained results, rather than as variables subject to direct empirical verification.

sociodemographic characteristics of the study group also provide an important context for interpreting the obtained results. The analyzed sample was dominated by single or divorced individuals, which may indicate limited family and social support. The literature emphasizes that the availability of social support is an important factor in adapting to mental illness and may also influence the level of insight into the illness and therapeutic cooperation (23).

The study group was dominated by individuals with primary and secondary education, while only a small proportion had higher education, which may have a significant impact on the understanding of the disease, interpretation of medical information, and shaping attitudes towards treatment (24, 25). Analysis of the structure of professional activity showed that the “other” category included individuals on disability pensions, social benefits, or in other forms of professional inactivity, which may reflect the long-term consequences of mental illness on social and professional functioning (26). Furthermore, the wide age range of the study sample suggests that generational differences may influence experiences of the disease, the level of knowledge about mental health, and attitudes towards pharmacotherapy and psychiatric treatment (27).

Due to the limited sample size and uneven distribution of some categories of sociodemographic variables, it was decided not to include them as predictors in statistical analyses, as this could lead to unstable parameter estimates and an increased risk of interpretation errors.

The obtained results have significant clinical implications, particularly in the context of forensic psychiatry departments, where the treatment process is lengthy, compulsory, and burdened by additional legal factors. The moderate to high levels of illness acceptance demonstrated by the majority of patients, as well as the predominance of positive attitudes toward pharmacological treatment, indicate that, despite the difficult conditions of hospitalization, it is possible to achieve relatively stable psychological adaptation.

The obtained results also indicate the need to treat illness acceptance and attitudes toward treatment as distinct, yet complementary, areas of therapeutic intervention. Interventions aimed solely at improving insight or prolonging hospitalization may prove insufficient without concurrent work on patients’ subjective beliefs about pharmacological treatment. Future studies should consider longitudinal designs that include assessment of the AIS/DAI across different phases of the illness.

### 4.1 Limitations of the study

This study has several limitations that should be considered when interpreting the results. The observed relationships—or lack thereof—reflect only the status of the study population at a specific point in time during hospitalization and do not allow for an assessment of the dynamics of change over time.

Another limitation is the purposive selection of the sample and its limited size, which includes patients from forensic psychiatry departments. The specific nature of this population, related to long-term hospitalization and legal conditions of treatment, limits the generalizability of the results to other groups of psychiatric patients, particularly those treated on an outpatient basis or hospitalized in general psychiatric wards.

Additionally, excluding individuals with acute psychosis/mania, severe psychotic depression, severe negative symptoms, current suicidal tendencies, significant cognitive deficits, or those unable to provide informed consent biased the sample toward relatively clinically stable patients. This means that our results apply primarily to the forensic population in symptomatic compensation, rather than to the entire spectrum of forensic psychiatric patients.

It is also important to note the exclusive use of self-report instruments (AIS, DAI), which, despite their good reliability and validity, are susceptible to factors such as a tendency to provide socially acceptable responses, limited insight into the illness, or the participants’ current mental state. In forensic psychiatry, these factors may be particularly important, especially in the context of the perceived consequences of participating in the study.

Another limitation is the lack of consideration of other potentially important moderating and mediating variables, such as the level of insight into the illness, the severity of psychopathological symptoms, the experience of adverse drug reactions, the therapeutic relationship, and the scope of psychoeducational interventions. These factors, as current research indicates, may significantly influence both illness acceptance and attitudes toward pharmacological treatment.

Finally, the analysis of length of hospitalization was based on broad time categories, which, although methodologically justified due to the size of the subgroups, may have limited the sensitivity to detect subtle differences between patients at different stages of treatment.

Despite the indicated limitations, the study provides valuable data on the psychological functioning of patients hospitalized in forensic psychiatry departments and may constitute a starting point for further, in-depth longitudinal studies, taking into account a wider spectrum of clinical and psychosocial variables.

## 5 Conclusions

Patients hospitalized in forensic psychiatry departments are characterized by a varied, most often medium or high level of acceptance of the disease and a predominantly positive attitude towards pharmacological treatment, with no statistically significant correlations between acceptance of the disease and attitudes towards pharmacotherapy and no effect of the length of hospitalization on these attitudes, which indicates the need to individualize therapeutic interventions regardless of the duration of hospital stay.

## Data Availability

The original contributions presented in the study are included in the article/supplementary material. Further inquiries can be directed to the corresponding author.
